# Natural and Technical Phytoremediation of Oil-Contaminated Soil

**DOI:** 10.3390/life13010177

**Published:** 2023-01-07

**Authors:** Leonid Panchenko, Anna Muratova, Ekaterina Dubrovskaya, Sergey Golubev, Olga Turkovskaya

**Affiliations:** Institute of Biochemistry and Physiology of Plants and Microorganisms, Saratov Scientific Centre of the Russian Academy of Sciences (IBPPM RAS), 13 Prospekt Entuziastov, 410049 Saratov, Russia

**Keywords:** oil hydrocarbons, technical phytoremediation, natural phytoremediation, *Medicago sativa*, *Lolium perenne*, soil microorganisms

## Abstract

Natural and technical phytoremediation approaches were compared for their efficacy in decontaminating oil-polluted soil. We examined 20 oil-contaminated sites of 800 to 12,000 m^2^ each, with different contamination types (fresh or aged) and levels (4.2–27.4 g/kg). The study was conducted on a field scale in the industrial and adjacent areas of a petroleum refinery. Technical remediation with alfalfa (*Medicago sativa* L.), ryegrass (*Lolium perenne* L.), nitrogen fertilizer, and soil agrotechnical treatment was used to clean up 10 sites contaminated by oil hydrocarbons (average concentration, 13.7 g/kg). In technical phytoremediation, the per-year decontamination of soil was as high as 72–90%, whereas in natural phytoremediation (natural attenuation with native vegetation) at 10 other oil-contaminated sites, per-year decontamination was as high as that only after 5 years. Rhizodegradation is supposed as the principal mechanisms of both phytoremediation approaches.

## 1. Introduction

After nearly three decades of close study and application, phytoremediation has proven a sustainable, cost-effective, environmentally benign, and highly socially acceptable technology [1,2,3,4]. The range of pollutants treated by phytoremediation is wide and includes organic (petroleum hydrocarbons, pesticides, and antibiotics) and inorganic (heavy metals and radionuclides) pollutants [3,4]. Despite its limitations, such as the long processing time and the dependence on climatic conditions and plant competence toward certain pollutants [5,6,7], phytoremediation has undoubted advantages, such as low cost and the possibility of restoring large areas in situ [4,7,8]. For these reasons, phytoremediation is used widely in the eco-management of petrochemical enterprises. In some countries, including Russia and the USA, the use of plants and associated microorganisms for the cleanup of oil-contaminated areas is regulated by specific documents [9,10].

Phytoremediation is based on the natural recycling and utilization of pollutants present in the plant root zone. These processes are affected by root exudates and the metabolic activity of the soil microbiota. In response to environmental pollution by petroleum hydrocarbons, plants activate antioxidant enzymes [11,12,13]. Some of these enzymes (e.g., peroxidases, which have a wide substrate range) are present in root exudates and are implicated in the degradation of organic pollutants both *in planta* and *ex planta* [14,15,16,17]. Other root exudate components, such as organic acids, carbohydrates, and flavonoids, function as inducers of microbial metabolic activity or substrates for microbial growth in the plant root zone [18,19]. The plant promotion of soil microbial growth and the pollutant induction of microbial degradative activity constitute the main pathway for the soil degradation of petroleum hydrocarbons, rhizodegradation, which is the key mechanism of the phytoremediation of oil-contaminated soils [20,21,22].

The knowledge that has accumulated on the fundamentals of phytoremediation leads to the following question: Is natural vegetation in a contaminated area sufficient for soil rehabilitation, or is it necessary to resort to agrotechnical measures such as fertilization or special plant growing? Obviously, the duration of remediation will be different in either case. The decision in favor of natural or technical phytoremediation would be influenced by considerations such as the level of pollution (severe pollution hinders the growth of native vegetation), the period needed to make soil suitable for recultivation, and the suitability of other techniques (depending on the characteristics of the economic exploitation of the contaminated area). In this context, it is important to understand the difference between natural and technical phytoremediation, so that recultivation results could be predicted.

We compared the efficacy of natural versus technical phytoremediation of oil-contaminated soil in the industrial and adjacent areas of a petroleum refinery. In this study, the term “natural phytoremediation” means natural processes resulting in the cleanup or attenuation of oil pollution of soil covered with natural vegetation. The term “technical phytoremediation” means special agrotechnical remediation measures, such as soil tilling, soil fertilization, and the planting and watering of remediating plants. The progress of phytoremediation was assessed by measuring the vegetation cover, the oil concentration, the available nitrogen content, and the soil microbial content.

## 2. Materials and Methods

### 2.1. Monitoring of Oil-Contaminated Areas

The areas of study are located in the steppe zone of Russia’s Middle Volga region. The industrial and adjacent areas of the local petroleum refinery were monitored for 5 years. The total inspected area was about 340 ha and was divided into 297 sites. The soils of the surveyed area were based on shallow chernozems (WRB: Mollic Vertisols Eutric or FAO: Mollic Vertisols). The soils had a pH of 7.0–7.8, and their buffering capacity was high. The content of total organic carbon ranged from 1.5 to 6%; that of NO_3_, from 4 to 16 mg/kg; that of NH_4_, from 14 to 30 mg/kg; and that of P_2_O_5_, from 84 to 120 mg/kg.

During inspection, 20 oil-contaminated sites were identified. The predominant plant inhabitants, the foliage projective cover, and the soil content of total petroleum hydrocarbons (TPHs) were determined. The foliage projective cover (percentage of ground area occupied by the vertical projection of foliage) was determined by using the Ramenskiy mesh, as described [23]. Ramenskiy mesh is a small plate in which a rectangular 2 × 5-cm hole is made and is divided into 10 square cells of 1 cm^2^ each by using a thin wire. By looking at the herbage through the mesh hole, we determined how many 1 cm^2^ cells could be attributed to the foliage projection and how many to the uncovered soil surface, seen through the herbage. The plant species were counted and identified, as reported earlier [24].

### 2.2. Phytoremediation Approaches

As a result of monitoring, we chose 10 oil-contaminated sites that had a satisfactory (>20%) foliage projective cover. These were observed for a few years to evaluate the rate of natural phytoremediation. Once a year, we determined the soil content of TPHs, N-NO_3_, and N-NH_4_; the number of microorganisms; and the number of plants inhabiting the sites.

The other 10 sites were used for technical phytoremediation, which included soil tilling, soil fertilization, and planting and watering of remediating plants (Table 1). We used a mixture of legume and cereal plants. The principal remediating plants were alfalfa (*Medicago sativa* L.) and ryegrass (*Lolium perenne* L.). Preliminary laboratory and pot experiments had led us to find that both species were effective at reducing the content of all hydrocarbon fractions in oil-sludge-contaminated soil [25]. Watering and soil rotary cultivation are trivial soil-remediation approaches.

Soil was sampled before plant sowing, after 3 and 12 months, and then annually. Samples were taken at several local points from a depth of 5–15 cm by using the envelope method. Mixed samples (~1.0 kg), combined from 5–8 local point samples from each site, were divided into replications (*n* ≥ 3) to measure the soil content of TPHs, N-NO_3_, N-NH_4_, and the number of microorganisms.

### 2.3. Soil Chemical Analysis

The soil content of TPHs was measured by gravimetric analysis, as described in the federal regulatory document [26]. The method is based on the extraction of oil products with chloroform from air-dried soil, separation of polar compounds by liquid chromatography after solvent replacement with hexane, and measurement by gravimetric analysis. A soil sample (~30 g dry weight) was placed into a 150 mL flask, and 10–15 mL of chloroform was added. For extraction, the flask was shaken for 5 min, and the chloroform extract was decanted and filtered through ashless paper (pore diameter, 8–12 µm) into a beaker. The procedure was repeated 3–4 times until the extract was decolorized. The extracts were combined and evaporated. The residue was redissolved in 5–10 mL of hexane and was passed through a column (120–150 mm × 10 mm) containing 6–8 g of activated alumina. The eluate was collected in preweighed beakers. After the solvent evaporated, the beakers were reweighed, and the TPH content was calculated. All soil samples were analyzed in triplicate. The tentative allowable concentration of TPHs in nonindustrial soil, as recommended at the regional level, is 1.0 g/kg [27].

Soil nitrates and water-soluble ammonium were measured by standard photocolorimetric methods [28,29]. The determination of nitrates included the following steps: extraction of nitrates from soil with a potassium chloride solution, reduction of nitrates to nitrites with hydrazine in the presence of copper as a catalyst, and photometric measurement of the colored diazo compound formed. The determination of exchangeable ammonium included the following steps: extraction of exchangeable ammonium from soil with a potassium chloride solution, generation of a colored indophenol compound formed by the interaction of ammonium with hypochlorite and sodium salicylate in an alkaline medium, and photometry of the colored solution.

### 2.4. Soil Microbiological Analysis

Total cultivable heterotrophic microorganisms (THMs) were enumerated by the plating dilution technique by using a beef extract agar medium. The membrane filter technique and Bushnell and Haas’s medium [30] with sterilized crude oil were used to enumerate hydrocarbon-oxidizing microorganisms (HOMs), as described previously [25].

### 2.5. Statistics

Data were processed by calculating the means of at least three replicates. Standard deviations (SD) and confidence intervals were used at *p* ≤ 0.05. Spearman’s rank correlation analysis was used to find the relations between the measured parameters. Statistica 13 (TIBCO Software Inc. 2017, Statsoft Russia, Palo Alto, CA, USA) and Microsoft Excel 2007 (Microsoft, Redmond, WA, USA) software were used for analysis.

## 3. Results

### 3.1. Monitoring of Oil-Contaminated Areas

Table 2 lists the principal characteristics of the oil-contaminated sites. The average TPH content in the soil of the sites was 13.7 g/kg, ranging from 4.2 to 27.4 g/kg in the mixed samples taken from five locations at each site. The foliage projective cover at sites S-1–S-10 was about 20–70%. With the allowance for the economic purpose of these sites, they were considered suitable for natural attenuation (natural phytoremediation). The foliage projective cover at sites S-11–S-20 was too low, and the contaminated areas were not too large. These sites were considered suitable for remediation by the planting of effective remediating plants (technical phytoremediation).

### 3.2. Natural Phytoremediation

**Figure 1:**
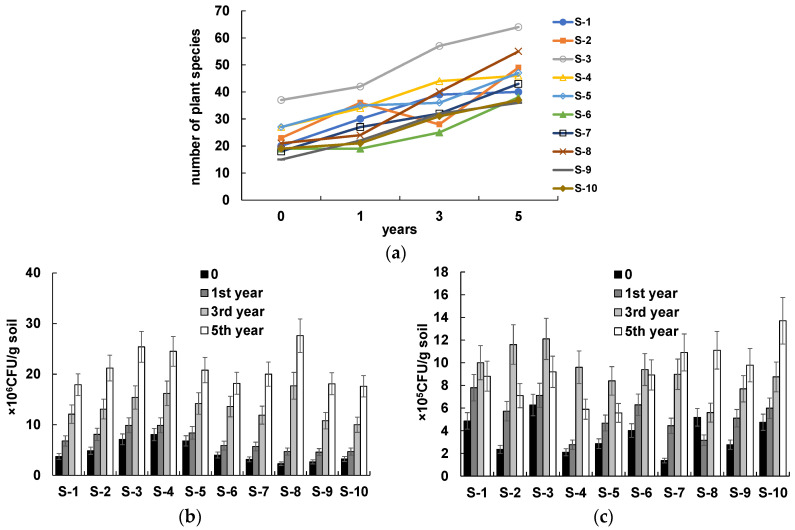
Numbers of plant species and culturable microorganisms in the oil-contaminated soil subjected to natural phytoremediation: (**a**) numbers of plant species; (**b**) THM number; and (**c**) HOM number.

As a result of monitoring, oil-contaminated sites S-1–S-10, which had a satisfactory foliage projective cover, were observed for a few years to evaluate the rate of natural phytoremediation. Once a year, we determined the soil content of TPHs and available nitrogen; the numbers of THMs and HOMs; and the number of plants inhabiting the sites. Figure 1 shows the time course of changes in the diversity of plant species and in the abundance of culturable microorganisms in the soil subjected to natural phytoremediation. As can be seen, within 5 years, the diversity of plant species increased at all sites surveyed (Figure 1a). In total, 203 species were identified, with the predominant species being *Elytrigia repens*, *Bromus squarrosus*, *Calmagrostis epigeios*, *Poa pratensis*, *Polygonum aviculare*, *Euphorbia virgata*, *Medicago falcata*, *Cichorium intybus*, and *Artemisia austriaca* and *absinthium*.

The increases in plant species diversity and the vegetation cover in plots S-1–S-10 were accompanied by increases in the numbers of soil microorganisms (Figure 1b,c). At all sites, the THM number increased steadily for 5 years (Figure 1b). The HOM number peaked in the third year, after which it decreased at the sites at which the TPH content decreased to less than 1.0 g/kg (sites S-1–S-6; Figure 1c). By contrast, the HOM number at sites S-7–S-10 continued to increase.

With increased foliage projective cover and species diversity, the content of ammonium nitrogen gradually increased from 12 to 28 mg/kg at the beginning of the observation period to 26–38 mg/kg after 5 years (Table 3). The nitrate content also increased from 2 to 4 mg/kg to 3–6 mg/kg in the same period. No unidirectional dynamics were found in the phosphorus content (data not shown), which ranged from 29 to 123 mg/kg throughout the observation period. The soil pH remained close to neutral, with a gradual increase of 6.8 to 7.3 within 5 years.

The increase in plant diversity was accompanied by a reduction in the soil TPH content. The average initial content of TPHs at sites S-1–S-10 was about 13.0 g/kg. Natural phytoremediation decreased the average TPH content to 7.8, 4.8, and 1.8 g/kg in the first, third, and fifth years, respectively.

**Figure 2:**
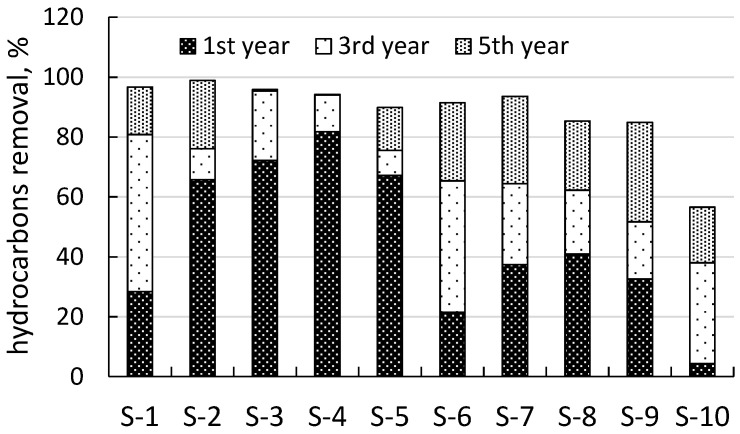
Natural phytoremediation rates recorded for the five years of treatment.

The phytoremediation rate differed markedly between sites (Figure 2), possibly because of the nature of oil pollution (Table 2). In the first year, the phytoremediation rate was highest at freshly polluted sites (S-2–S-5) and minimal at the sites with a history of oil pollution (S-1, S-6, and S-10). The decontamination of these latter sites was more effective in the subsequent years. Notably, during the first year, the phytoremediation efficiency ranged widely from 4% (at S-10) to 82% (at S-4).

In the third year, the TPH content at sites S-3 and S-4 already was within the maximum permissible limits (<1000 mg/kg [27], Table 3). In the fifth year, the same picture emerged at four more sites (S-1, S-2, S-5, and S-6). Among these sites, S-2 had a high initial TPH content (about 19 g/kg) and eventually showed the highest phytoremediation rate (98.9%).

### 3.3. Technical Phytoremediation

The technical phytoremediation efficiency was evaluated after three months and then after one, three, and five years of treatment. During technical phytoremediation, the foliage projective cover at the worst polluted sites increased from 20 to 40% in the first year. As early as next year, sites S-11–S-20 had native plants such as *Melilotus officinalis* and *Agropyron cristatum*, as well as cultivated plants such as *Medicago sativa* and *Lolium perenne*. In the third year, the vegetation cover included both introduced and native plant species, and the number of the latter was much larger than that of the former.

**Figure 3:**
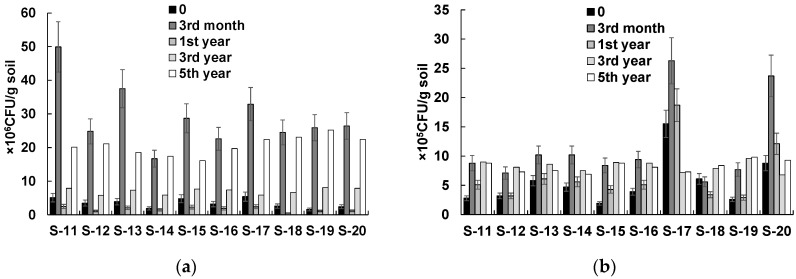
Numbers of culturable microorganisms in the oil-contaminated soil subjected to technical phytoremediation: (**a**) THM number; (**b**) HOM number.

Figure 3 and Table 4 show the results of the soil microbial and chemical analyses for technically remediated sites S-11–S-20. One can see that in the first three months of treatment, soil microorganisms were activated (Figure 3). The number of THMs increased by 10 times in response to nitrogen fertilization. The number of HOMs increased as well. A year later, however, the number of THMs dropped sharply. The soil content of available nitrogen also decreased (Table 4). The increased content of available nitrogen in the third month of treatment was associated with the application of nitrogen fertilizer.

The average initial content of TPHs at sites S-10–S-20 was about 14.4 g/kg. Technical phytoremediation decreased the average TPH content to 2.5, 1.9, and 1.1 g/kg in the first, third, and fifth years, respectively.

**Figure 4:**
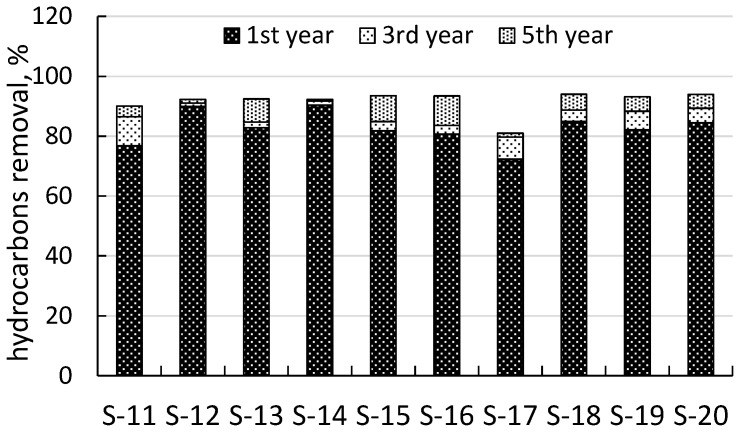
Technical phytoremediation rates recorded for the five years of treatment.

At all sites, technical phytoremediation was noticeably effective as early as after a year of treatment, regardless of the history and level of oil pollution (Figure 4).

The average phytoremediation rate was 82%, ranging from 72 to 90% at different sites.

## 4. Discussion

Which method is chosen for the recultivation of oil-contaminated soil is primarily determined by the type and properties of the soil, the kind and concentration of the pollutant, the area of the land, the natural conditions, and the economic purpose of the contaminated area. All these factors may limit the period and scale of use of the chosen technology. Comparative studies of bioremediation techniques such as natural attenuation, biostimulation, bioaugmentation, mycoaugmentation, and phytoremediation have been conducted mainly in the laboratory [31,32,33], and few have been conducted in the field [34,35]. Many researchers agree that although phytoremediation is inferior to other bioremediation methods in terms of the decontamination rate [7,31], it is sustainable, cheap, and effective in terms of the completeness of decontamination [7,31,32]. Actual large-scale field phytoremediation trials are few [36,37,38,39,40,41], and we did not find comparisons of the efficacy of natural versus introduced vegetation in the restoration of oil-contaminated soils. In this sense, our results may be of some interest.

In this five-year study, the establishment of a vegetation cover was a fundamental component of phytoremediation. The oil-contaminated areas were naturally revegetated. The increase in species diversity at the sites with a history of oil pollution was higher (219% on average, 10 sites) than at the freshly polluted sites (194% on average, 10 sites) (Figure 2), possibly owing to the toxicity of not yet volatilized light oil hydrocarbons [42].

Correlation analysis confirmed that as the contaminated areas developed a vegetation cover, the total number of microorganisms, including oil degraders, increased (*r_s_* = 0.77; *p* < 0.05). This dependence can be explained by the rhizosphere effect, i.e., stimulation by plant root exudates of the abundance and activity of the root zone microorganisms [19,43]. Soil microorganisms, particularly oil-degrading bacteria, are major actors involved in the removal of petroleum hydrocarbons from the environment [20,21,44].

The numbers of THMs and HOMs changed differently between natural and technical phytoremediation treatments. In natural phytoremediation, the microbial numbers increased gradually from 4.2 to 21.1 × 10^6^ CFU/g soil (THMs) and from 3.3 to 9.1 × 10^5^ CFU/g soil (HOMs) (average figures for 10 sites). The THM and HOM numbers peaked in the fifth year of study as the contaminated sites developed a vegetation cover. Technical phytoremediation, including soil agrotechnical treatment, stimulated the development of a soil indigenous microflora and accelerated the natural revegetation of the sites, ultimately leading to the cleaning and ecological restoration of the soil. The microbial numbers at the sites subjected to technical phytoremediation increased by 10 times (from 3.1 to 29.0 × 10^6^ CFU/g of soil (THMs) and from 5.0 to 11.7 × 10^5^ CFU/g of soil (HOMs) (average values for 10 sites)), and they were highest after nitrogen fertilizer had been applied. However, as early as a year after the start of remediation, the microbial numbers decreased to the initial level (to 1.7 × 10^6^ CFU/g of soil (THMs) and to 6.8 × 10^5^ CFU/g of soil (HOMs) (average values for 10 sites)). A significant inverse correlation was found between the THM number and the pollutant content in the soil (*r_s_* = −0.59; *p* < 0.05). Gradually, the number of THMs at the sites subjected to technical phytoremediation increased as the ecosystem was restored, and in the fifth year of study, it was comparable to the microbial number in the soil of the sites subjected to natural phytoremediation.

It is known that soil nitrogen fertilization causes a priming effect—an increase in the number and activity of microorganisms and an increase in the decomposition of organic matter [45]. This is what we observed during technical phytoremediation. Previous work has shown that the application of nitrogen fertilizer promotes the biodegradation of petroleum hydrocarbons in soil [46,47]. Our study revealed a close correlation between the content of ammonium nitrogen and the efficiency of phytoremediation at each stage (first year, *r_s_* = 0.57; third year, *r_s_* = 0.76; and fifth year, *r_s_* = 0.50; *p* < 0.05). The explosive growth in the number of microorganisms resulted in the high degradation of petroleum hydrocarbons in the same period, regardless of the concentration and age of the pollution. By the end of the first year, the utilization of organic matter and the depletion of nutrient nitrogen, intensified by competition for nitrogen from the introduced plants, led to a decrease in the number of soil microorganisms, but in this period, the soil had already been clean. We believe that this is how the events associated with technical phytoremediation occurred under the described conditions.

In natural phytoremediation, decontamination proceeded more slowly than in technical phytoremediation. However, it was stably supported by the developing vegetation. As a vegetation cover developed, the content of available nitrogen increased slowly. This was confirmed by the significant correlation found between the area of the foliage projective cover and the soil content of NO_3_ (*r_s_* = 0.60; *p* < 0.05). As plant roots grow, they change soil conditions, contributing to an increase in the number and activity of soil microorganisms and to the degradation of petroleum hydrocarbons [48]. Thus, the effectiveness of natural phytoremediation is largely determined by the state of the vegetation cover. According to Drugov and Rodin [27], the natural decontamination rates for oil-contaminated soils of different natural biomes but of the same oil-pollution level (5000 mg/kg) are evaluated for the following periods: up to 5 years (high natural decontamination rate), up to 10 years (medium natural decontamination rate), and up to 30 years and more (low natural decontamination). According to this classification, the rate of natural decontamination at the naturally vegetated sites included in this study is high, exceeding 90% in five years.

This research was conducted in the grounds of a petroleum refinery, and we were able to compare the economic costs of different phytoremediation approaches. On the basis of the cost of a year-long technical phytoremediation project, the reclamation of 1 ha was estimated to cost RUB 1 million (~$14,300/ha or $1.4/m^2^). Natural phytoremediation did not require costs other than annual costs, including those of mowing, the collection and utilization of plant biomass in both remediated and nonremediated areas around the refinery, and the monitoring of the refinery grounds to find contaminated sites. The cost of a year-long monitoring of the refinery grounds was RUB 140,000/ha (~$2000/ha or $0.2/m^2^).

## 5. Conclusions

The remediation efficiency for the oil-contaminated sites in the industrial and adjacent areas of the petroleum refinery was high enough in both technical and natural phytoremediation. However, the rate of technical phytoremediation was higher than that of natural phytoremediation, permitting an acceptable cleanup level to be attained in a shorter period. In technical phytoremediation, including the tilling of soil and the planting and watering of *M. sativa* and *L. perenne*, the per-year decontamination of soil from oil hydrocarbons was as high as 72–90%. The efficiency of natural phytoremediation (natural attenuation of soil with the native vegetation cover) was as high as that only after 5 years of treatment. Thus, the high rate and efficiency of technical phytoremediation as a soil rehabilitation approach was confirmed. However, the possibilities of natural phytoremediation, a cost-free process that takes more time than does technical phytoremediation but yields the same results, should not be underestimated. When time permits, the duration of natural phytoremediation may be no bar to its being chosen for the rehabilitation of contaminated areas.

## Figures and Tables

**Table 1:** Procedures used for technical phytoremediation.

Procedure	Quantity	Period
Soil milling	WM1100BE walk-behind tractor (Weima China) Processing depth, 25–30 cm	Before remediation
Fertilization: Azophoska mineral fertilizer (NPK, 22:11:11)	3 kg/100 m^2^ × 2	Before and 1.5 months after sowing
Watering	30 L/m^2^	One day before sowing
Sowing: *Medicago sativa* *Lolium perenne*	0.4 kg/100 m^2^ 0.5 kg/100 m^2^	April–May
Rolling seedlings		After sowing
Soil sampling	One mixed sample was made from five to eight local point samples taken from a depth of 5–15 cm at each site	Before sowing, after 3, 12 months, then annually
Watering	20 L/m^2^ × 2	Before sowing, and 3 weeks after seedling appearance

**Table 2:** Characterization of the oil-contaminated sites.

Site No.	Characterization	Area (m^2^)	Foliage Projective Cover (%)	TPHs (g/kg)
S-1	Former oil sludge pit (aged oil pollution)	12,000 *	25–30	5.3 ± 0.3
S-2	Fresh and aged oil spills near oil pipelines	2800	65–70	18.8 ± 2.6
S-3	Fresh and aged oil spills near oil pipelines	5000	50–55	4.2 ± 0.2
S-4	Fresh and aged oil spills near oil pipelines	4800	60–65	6.6 ± 0.5
S-5	Fresh and aged oil spills near oil pipelines	1400	50–55	6.8 ± 0.4
S-6	Aged subsurface oil pollution near oil pipelines and tanks	2100	25–30	6.1 ± 0.3
S-7	Fresh and aged oil spills near oil pipelines	16,200	40–45	24.5 ± 2.3
S-8	Aged subsurface oil pollution near oil pipelines and tanks	5100	35–40	12.6 ± 0.7
S-9	Aged subsurface oil pollution near oil pipelines	1800	40–45	27.4 ± 2.5
S-10	Former oil sludge pit (subsurface aged oil pollution)	10,000	20	18.2 ± 1.0
S-11	Fresh and aged oil spills near oil pipelines and tanks	800	<20	10.8 ± 0.5
S-12	Fresh and aged oil spills near oil pipelines and tanks	850	<20	8.4 ± 0.8
S-13	Fresh and aged oil spills near oil pipelines and tanks	820	<20	16.6 ± 1.8
S-14	Fresh and aged oil spills near oil pipelines and tanks	860	<20	9.4 ± 0.5
S-15	Fresh and aged oil spills near oil pipelines and tanks	920	<20	15.6 ± 1.4
S-16	Fresh and aged oil spills near oil pipelines and tanks	800	<20	14.8 ± 0.8
S-17	Former oil sludge pit (aged oil pollution)	1200	<20	5.5 ± 0.3
S-18	Former oil sludge pit (aged oil pollution)	1200	<20	19.4 ± 0.9
S-19	Former oil sludge pit (aged oil pollution)	1100	<20	27.1 ± 1.4
S-20	Former oil sludge pit (aged oil pollution)	800	<20	16.3 ± 0.7

* Values are means ± SD (*p* ≤ 0.05) of at least three samples.

**Table 3:** Time course of the content of available nitrogen and TPHs in the oil-contaminated soil subjected to natural phytoremediation.

Site No.	N-NH_4_^+^ (mg/kg)				N-NO_3_^−^ (mg/kg)				TPHs (g/kg)			
	Initial	1st Year	3rd Year	5th Year	Initial	1st Year	3rd Year	5th Year	Initial	1st Year	3rd Year	5th Year
S-1	12.1 ± 0.6 *	18.2 ± 1.0	26.1 ± 1.7	27.9 ± 1.3	2.4 ± 0.2	2.3 ± 0.1	3.5 ± 0.2	3.5 ± 0.1	5.3 ± 0.3	3.8 ± 0.3	1.0 ± 0.1	0.2 ± 0.0
S-2	25.0 ± 0.1	29.3 ± 1.3	31.7 ± 1.5	37.3 ± 1.8	4.5 ± 1.9	5.2 ± 0.3	6.3 ± 0.3	6.5 ± 0.3	18.8 ± 2.6	6.4 ± 0.4	4.5 ± 0.3	0.2 ± 0.0
S-3	28.2 ± 1.5	30.9 ± 1.4	32.3 ± 1.4	36.7 ± 2.0	6.1 ± 0.3	5.4 ± 0.2	5.6 ± 0.2	6.1 ± 0.4	4.2 ± 0.2	1.2 ± 0.0	0.2 ± 0.0	0.2 ± 0.0
S-4	26.1 ± 1.2	31.3 ± 1.5	34.3 ± 1.5	35.9 ± 1.4	5.9 ± 0.4	6.2 ± 0.3	6.1 ± 0.4	6.1 ± 0.3	6.6 ± 0.5	1.2 ± 0.1	0.4 ± 0.0	0.4 ± 0.0
S-5	18.3 ± 1.0	26.2 ± 1.4	25.4 ± 1.6	29.1 ± 1.6	2.2 ± 0.1	4.8 ± 0.2	4.4 ± 0.3	5.2 ± 0.3	6.8 ± 0.4	2.2 ± 0.1	1.7 ± 0.1	0.7 ± 0.1
S-6	18.2 ± 0.9	26.8 ± 1.5	26.2 ± 1.2	31.3 ± 1.5	3.1 ± 0.1	5.3 ± 0.3	5.2 ± 0.3	5.1 ± 0.3	6.2 ± 0.3	4.8 ± 0.3	2.1 ± 0.1	0.5 ± 0.1
S-7	16.4 ± 0.7	23.8 ± 1.1	25.1 ± 1.2	32.2 ± 1.5	2.5 ± 0.1	3.6 ± 0.4	4.8 ± 0.2	5.5 ± 0.5	24.5 ± 2.3	15.3 ± 0.9	8.7 ± 0.6	1.6 ± 0.1
S-8	15.0 ± 0.7	20.3 ± 0.9	21.9 ± 1.4	31.4 ± 1.7	5.1 ± 0.2	5.2 ± 0.2	5.4 ± 0.3	6.3 ± 0.3	12.6 ± 0.7	7.4 ± 0.3	4.7 ± 0.3	1.8 ± 0.1
S-9	14.2 ± 0.8	181 ± 1.1	21.7 ± 1.0	26.1 ± 1.8	5.3 ± 0.3	5.4 ± 0.3	5.7 ± 0.4	5.6 ± 0.3	27.4 ± 2.5	18.5 ± 1.5	13.2 ± 0.7	4.1 ± 0.3
S-10	1.3 ± 0.2	11.4 ± 0.5	24.4 ± 1.6	30.8 ± 1.4	2.6 ± 0.1	4.7 ± 0.2	5.5 ± 0.2	6.0 ± 0.5	18.2 ± 1.0	17.4 ± 0.9	11.3 ± 0.6	7.9 ± 0.6

* Values are means ± SD (*p* ≤ 0.05) of at least three samples.

**Table 4:** Time course of the content of available nitrogen and TPHs in the oil-contaminated soil subjected to technical phytoremediation.

Site No.	N-NH_4_^+^ (mg/kg)			N-NO_3_^−^ (mg/kg)			TPHs (g/kg)			
	Initial	3rd Month	1st Year	Initial	3rd Month	1st Year	Initial	1st Year	3rd Year	5th Year
S-11	23.5 ± 1.1 *	37.2 ± 1.8	29.1 ± 1.7	1.8 ± 0.1	17.5 ± 0.8	5.4 ± 0.2	10.8 ± 0.5	2.5 ± 0.1	1.5 ± 0.1	1.1 ± 0.1
S-12	24.1 ± 1.3	37.5 ± 2.0	28.5 ± 1.4	3.4 ± 0.2	20.2 ± 1.0	4.3 ± 0.3	8.4 ± 0.8	0.9 ± 0.1	0.8 ± 0.1	0.7 ± 0.1
S-13	19.4 ± 0.9	38.4 ± 1.8	28.2 ± 1.4	2.7 ± 0.1	18.4 ± 1.1	5.2 ± 0.2	16.6 ± 1.8	2.9 ± 0.2	2.6 ± 0.1	1.3 ± 0.1
S-14	24.5 ± 1.1	40.6 ± 2.0	27.4 ± 1.5	2.3 ± 0.1	24.0 ± 1.1	6.5 ± 0.3	9.4 ± 0.5	0.9 ± 0.1	0.8 ± 0.1	0.7 ± 0.1
S-15	22.1 ± 1.4	37.1 ± 1.9	29.0 ± 1.3	1.6 ± 0.2	21.0 ± 0.9	6.1 ± 0.4	15.6 ± 1.4	2.9 ± 0.1	2.4 ± 0.1	1.0 ± 0.1
S-16	20.3 ± 1.2	39.5 ± 1.6	26.2 ± 1.2	1.9 ± 0.2	25.4 ± 1.2	5.0 ± 0.2	14.8 ± 0.8	2.9 ± 0.2	2.4 ± 0.1	1.0 ± 0.1
S-17	16.2 ± 0.7	22.5 ± 1.1	16.4 ± 1.1	3.7 ± 0.1	15.4 ± 0.9	1.7 ± 0.1	5.5 ± 0.3	1.5 ± 0.2	1.1 ± 0.1	1.1 ± 0.1
S-18	15.5 ± 0.7	25.2 ± 1.5	16.3 ± 1.2	4.2 ± 0.2	15.5 ± 0.7	1.8 ± 0.2	19.4 ± 0.9	2.9 ± 0.1	2.2 ± 0.1	1.2 ± 0.1
S-19	21.1 ± 1.5	35.1 ± 1.6	20.0 ± 0.9	1.6 ± 0.1	11.8 ± 0.6	2.4 ± 0.2	27.1 ± 1.4	4.9 ± 0.2	3.2 ± 0.2	1.9 ± 0.2
S-20	28.4 ± 1.4	42.3 ± 2.1	19.5 ± 1.0	2.4 ± 0.1	16.5 ± 0.7	2.9 ± 0.2	16.3 ± 0.7	2.5 ± 0.1	1.8 ± 0.1	1.0 ± 0.1

* Values are means ± SD (*p* ≤ 0.05) of at least three samples.

## Data Availability

Not applicable.
